# Isolation and genomic analysis of seven phages active against carbapenem-resistant *Klebsiella pneumoniae*

**DOI:** 10.1128/mra.00768-25

**Published:** 2025-11-18

**Authors:** Luz Cuello, Krupa Parmar, Adeline Supandy, Daria Van Tyne, Robin Patel

**Affiliations:** 1 Mayo Clinic Graduate School of Biomedical Sciences, Virology and Gene Therapy Track 4352 , Rochester, Minnesota, USA; 2 Division of Clinical Microbiology, Mayo Clinic 4352 , Rochester, Minnesota, USA; 3 Division of Infectious Diseases, University of Pittsburgh School of Medicine 12317 , Pittsburgh, Pennsylvania, USA; 4 Center for Evolutionary Biology and Medicine, University of Pittsburgh School of Medicine 12317 , Pittsburgh, Pennsylvania, USA; 5 Division of Public Health, Infectious Diseases, and Occupational Medicine, Mayo Clinic Minnesota 4352 , Rochester, Minnesota, USA; Loyola University Chicago, Chicago, Illinois, USA

**Keywords:** bacteriophages, *Klebsiella*, phylogenetic analysis, isolation, genome analysis

## Abstract

The genomic sequences of seven phages isolated from municipal sewage in Rochester, Minnesota, USA, using *Klebsiella pneumoniae* strains (*bla*_KPC_ carriers) as hosts are reported. These phages are lytic and belong to the *Ackermannviridae, Drexlerviridae*, and *Zobellviridae* families. No known antimicrobial resistance or virulence genes were found within their genomes.

## ANNOUNCEMENT

Carbapenem-resistant *Klebsiella pneumoniae* (CRKP) are Gram-negative pathogens, which can cause difficult-to-treat infections, especially in hospitalized and immunocompromised patients (1). CRKP poses a threat to public health due to increasing global antimicrobial resistance (AMR) and a stagnating pipeline for novel antibiotic discovery (2). Phages could provide an alternative option against these multidrug-resistant infections. Here, the isolation and characterization of seven lytic phages active against CRKP are reported; all phages were sourced from one public sewage sample (collected 22 November 2022, in Rochester, Minnesota, USA [44.06377, −92.46758], transported in a 2 L sterile glass bottle, and stored at 4°C).

Sewage was enriched as described (3) in tryptic soy broth (overnight incubation, 37°C), using individual *K. pneumoniae* clinical isolates (all *bla*_KPC_ carriers; *n *= 24 from the Antibacterial Resistance Leadership Group (ARLG) (4, 5), and *n *= 4 from the Clinical Microbiology Laboratory at Mayo Clinic). Single plaques were picked and passaged at least thrice using the double-agar overlay method (6, 7). Plaque-purified phage lysates (800 µL–1600 µL) were treated with DNAse I (2 U/µL) and RNAse A (10 mg/mL), and DNA was isolated using the Quick-DNA Viral Kit (Zymo Research), or the Maxwell® RSC Blood DNA Kit (Promega), following manufacturers’ instructions.

Sequencing libraries were prepared using the Illumina DNA Prep tagmentation kit and Illumina Unique Dual Indexes and sequenced on an Illumina NextSeq2000 platform using a 300-cycle flow cell kit to produce 2 × 150 bp paired-end reads. Raw reads were trimmed with Trimmomatic (8) (v. 0.39 + galaxy2) and quality-checked with FastQC (9) (v. 0.74 + galaxy1). Genomes were assembled using metaviralSPAdes (10) (v. 3.15.5 + galaxy2). Assemblies were quality-checked using Quast (11) (v. 5.2.0 + galaxy1) and annotated with Pharokka (12) (v. 1.3.2 + galaxy0). Genomes were screened for AMR and virulence genes using ABRicate (13) (v. 1.0.1). When possible, genome ends were determined with PhageTerm (14) (v. 1.0.12). Software tools were run in Galaxy (15, 16) with default parameters. Genome lengths and assembly statistics are reported in Table 1. Phage lifestyle predictions were determined using PhageAI (17). Phylogeny was established through NCBI Nucleotide BLAST searches and taxMyPhage (18). For each phage, the top 10 most similar complete phage sequences in NCBI (maximum score parameter) were used as input for construction of a phylogenetic tree using VICTOR (19). Phage morphology was assessed by negative staining transmission electron microscopy.

**TABLE 1:** Genetic characterization of the isolated phages^*a*^^,*b*^

Phage	Family	Genus	CRKP isolation host	Genome size (bp)	GC- content (%)	Raw reads (n)	Trimmed reads (n)	Coverage (×)	Contig (n)	N50	Genome termini type and position (+/−)	Closest NCBI hit (accession number)	Similarity to closest NCBI hit (%)
vB_KpnM-Zahir	*Ackermannviridae*	*Taipeivirus*	ARLG-3318	160,669	46.52	1,391,799	21,152	291.0	1	160,669	Redundant, (150,944/60,802)	UPM 2146 (NC_049472.1)	97.92
vB_KpnM-Bestiario	*Ackermannviridae*	*Taipeivirus*	ARLG-4653	157,565	47.43	1,793,429	23,322	386.0	2	157,565	Redundant, (14,945/30,939)	vB_KpnM_Trex_ER12 (PP738761.1)	98.31
vB_KpnS-Cronopio	*Drexlerviridae*	*Webervirus*	ARLG-3529	46,531	50.63	1,133,541	21,272	810.0	1	46,531	Redundant, multiple	vB_KpnD_ghw (PP374644.1)	94.47
vB_KpnS-Fama	*Drexlerviridae*	*Webervirus*	ARLG-3529	45,921	50.61	1,539,163	22,593	1127.4	1	45,921	Redundant, multiple	vB_KpnS_2811 (LR757892.1)	94.51
vB_KpnS-Tlon	*Drexlerviridae*	*Webervirus*	IDRL-10368	49,297	50.83	1,384,776	22,706	973.4	1	49,297	Redundant, multiple	Sin4 (NC_049847.1)	93.64
vB_KpnS-Uqbar	*Drexlerviridae*	*Webervirus*	IDRL-10368	49,542	50.77	1,169,891	20,051	823.1	1	49,542	Redundant, multiple	RCIP0056 (OR532850.1)	91.96
vB_KpnP-Asterion	*Zobellviridae*	Not established. Closest related genus is *Citrovirus*	ARLG-4382	48,578	45.80	1,107,662	26,996	790.5	1	48,578	Redundant, multiple	vB_KpnP_Klyazma (OP125547.1)	93.46

^*a*^ The 28 *Klebsiella pneumoniae* isolates used for phage isolation included 24 isolates from the Antibacterial Resistance Leadership Group (ARLG-3415-P, ARLG-4501, ARLG-4653, ARLG-4888, ARLG-4496, ARLG-4429-P, ARLG-3623-P, ARLG-4428-P, ARLG-3245, ARLG-3594-P, ARLG-3529, ARLG-3614-P, ARLG-4212, ARLG-4425-P, ARLG-4674, ARLG-4380, ARLG-7547-P, ARLG-4190, ARLG-3328, ARLG-3321, ARLG-3318-P, ARLG-4382, ARLG-4490-P, and ARLG-3375), and 4 from the Mayo Clinic Clinical Microbiology Laboratory (IDRL-10368, IDRL-10370, IDRL-10373, and IDRL-10376). All isolates except for IDRL-10373 were resistant to meropenem (minimum inhibitory concentration, ≥4µg/mL) by broth microdilution.

^*b*^ NCBI database accessed 7 May 2025.

**Fig 1:**
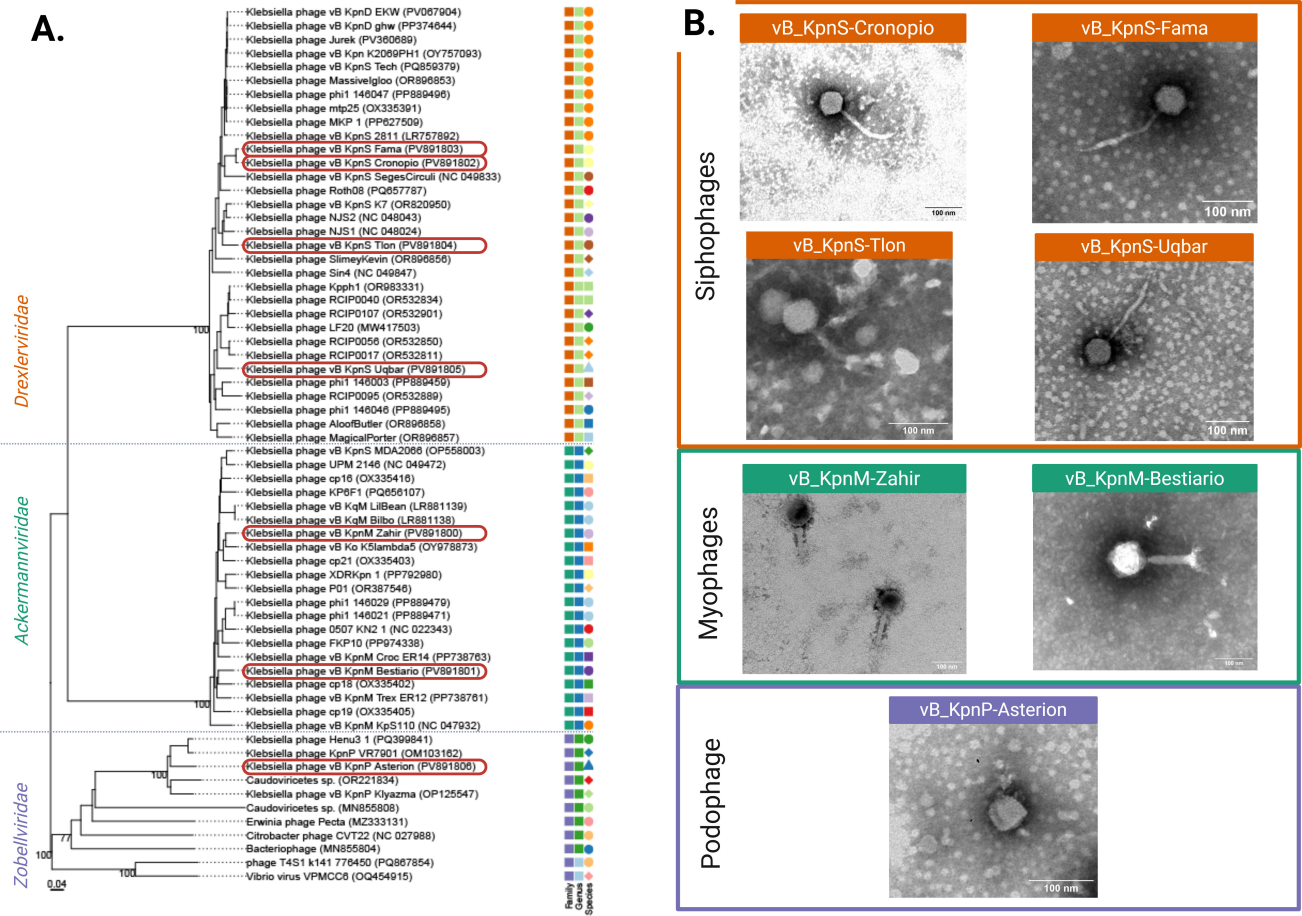
Morphologic and phylogenetic characterization of the isolated phages. (**A**) Phylogenetic tree showing the arrangement of taxa according to D0 equation (created using VICTOR). Isolated phages are highlighted. At the family level, squares indicate *Drexlerviridae* (dark orange), *Ackermannviridae* (dark green), and *Zobellviridae* (purple) clustering. At the genus level, squares indicate *Webervirus* (light green), *Taipeivirus* (blue), and *Citrovirus* (green) clustering. (**B**) Negative stain transmission electron micrographs of the isolated phages showing distinct morphologies. Colors correspond to phylogeny.

The seven isolated phages showed myo-, sipho-, and podophage morphotypes (*n *= 2, *n *= 4, and *n *= 1, respectively) (Fig. 1). Phylogenetic analysis results suggest clustering into the *Caudovirales* order, *Ackermannviridae* (vB_KpnM-Zahir and vB_KpnM-Bestiario)*, Drexlerviridae* (vB_KpnS-Cronopio, vB_KpnS-Fama, vB_KpnS-Tlon, and vB_KpnS-Uqbar), and *Zobellviridae* families (vB_KpnP-Asterion) (Fig. 1, Table 1). Genus identification was possible for 6/7 phages, which clustered into the *Taipeivirus* and *Webervirus* genera (Table 1). taxMyPhage analysis suggested that vB_KpnP-Asterion may represent a new species and genus. PhageTerm analysis showed redundant termini for all phage genomes; determination of genome termini position was only possible for vB_KpnM-Zahir and vB_KpnM-Bestiario (Table 1). All phages were scored ≥99.7% for a virulent lifestyle, with no known AMR or virulence genes. Thus, these newly isolated phages appear to be strictly lytic and show activity in CRKP strains, meriting further investigation as therapeutic alternatives.

## Data Availability

Phage genomic assemblies have been deposited in GenBank (accession numbers PV891800, PV891801, PV891802, PV891803, PV891804, PV891805, and PV891806). Raw sequencing reads are available under BioProject PRJNA1287990, Sequence Read Archive accessions SRR34427932, SRR34427931, SRR34427930, SRR34427929, SRR34427928, SRR34427927, and SRR34427926.
